# Irradiation Embrittlement in Grade-300 Steel Internals: A Comprehensive Description and Evaluation Approach

**DOI:** 10.3390/ma19194095

**Published:** 2026-09-24

**Authors:** Christian Robertson, Zitao Zeng

**Affiliations:** CEA, Service de Recherche en Matériaux et procédés Avancés, Université Paris-Saclay, 91191 Gif-sur-Yvette, France; theophile.zengzitao@foxmail.com

**Keywords:** steel, irradiation, poly-crystals, dislocation, slip, plasticity

## Abstract

**Highlights:**

This paper presents a comprehensive approach, focusing on intergranular crack initiation in grade 300 steel components exposed to neutron irradiation in water-cooled nuclear reactors. Our model is based on a detailed description of sub-grain plasticity mechanisms and available data on radiation-induced defect dispersions. More specifically:

- Plastic strain spreading is evaluated in each grain of a representative polycrystalline aggregate in terms of slip band thickness and spacing, depending on the applied loading level and the irradiation defect size and number density.
- The corresponding local grain boundary stress amplitude is calculated and rationalized, based on dimensionless factor «*R*» evolution.
- Factor «*R*» amplitude also depends on the grain boundary cohesive energy evolution, through local Δ*τ*_*c*_ correction.

**Abstract:**

Water-cooled nuclear reactors are equipped with internal structural elements made of grade 300 steels, which are subject to embrittlement over their operational lifetime. More specifically, typical internal components are simultaneously exposed to neutron irradiation and pressurized water at elevated temperatures, making them susceptible to intergranular crack initiation. Irradiation damage accumulation markedly affects plastic flow spreading, which is further coupled with strain localization effects. Concurrent exposure to the reactor coolant also contributes to grain-boundary configuration changes in terms of local chemical composition and the resulting tolerance to applied loading. Our approach for evaluating the material response integrates multiple physical mechanisms applicable to post-irradiation austenitic steels, including strain localization in the form of defect-depleted channels, dislocation pile-ups, and the loss of grain-boundary strength. The above time-dependent effects are evaluated and rationalized in terms of the evolution of the dimensionless factor «*R*», which depends on: (i) the applied loading level, (ii) the irradiation defect size and number density, and (iii) the local grain boundary cohesive energy evolution. The model predictions are consistent with an applied intergranular crack initiation stress of about 700 MPa after combined exposure to pressurized water and neutron irradiation up to 3 dpa at 300 °C.

## 1. Introduction

Internal PWR reactor components include bolts and baffle plates made of grade 300 austenitic steels, given their excellent corrosion resistance and high ductility across a broad range of utilization conditions. Internal components are subjected to the combined effect of high neutron flux and pressurized water at elevated temperature, and, therefore, are exposed to intergranular IASCC (Irradiation Assisted Stress Corrosion Cracking) hazards [1]. In practice, the post-irradiation response of grade 300 steel is affected by the dose-dependent accumulation of various types of radiation defects, including interstitial and vacancy loops and clusters [1,2]. The irradiation loop size generally increases with the irradiation temperature for a given irradiation dose [3]. In 300-series steels, loop number density typically increases with irradiation dose, reaching a saturation value of ~10^23^ m^−3^ for radiation doses between 3 and 10 dpa [4]. The defect dispersions generally influence plastic flow conditions, in the form of sub-grain slip bands [5,6,7]. Such bands sometimes appear as defect-depleted channels associated with surface slip markings [8,9,10,11,12].

Slip bands and markings gradually form during the deformation spreading, as the applied load activates the dislocation sources initially present in the material. Dose-dependent plastic flow localization directly affects the internal stress acting at the grain boundaries, depending on the applied load conditions [11,12]. Various modelling approaches have been recently developed in the context of post-irradiation damage prediction [13,14], including crystal plasticity (FEM) and fast Fourier transform (FFT) method calculations [15,16,17]. These and other similar (continuum-mechanics-based) attempts, however, contrast with the present approach, where plastic strain spreading is inherently discrete and dose-dependent.

Furthermore, significant chemical composition differences exist between cracked and uncracked grain boundary sites in post-irradiated specimens exposed to PWR conditions [18]. Cracked grain boundary sites actually present higher Cr and lower Ni concentrations than uncracked locations. The inner oxide at crack sites, on the other hand, is thinner and has a lower Cr content than at uncracked sites. Overall, Cr transport along grain boundaries and the protective role of inner oxides are found to play a critical role in crack susceptibility evolution, in addition to the other, above-described embrittlement contributions [18].

Accounting for all the details of grain boundary evolution is far too complex an endeavour, within the scope of the present article. A simplified yet insightful approach is proposed instead, where the main evolutions are described in terms of effective “grain boundary cohesive energy” changes, evaluated using the Read-Shockley sub-model. This approach helps evaluate the IASCC crack-initiation susceptibility of post-irradiated 300 steels using a limited set of supporting data and physical parameters.

The calculations prescribed in this manuscript, however, go beyond phenomenological methods by building around mechanisms relevant to irradiated austenitic steels, including localized deformation, defect-depleted channels, dislocation pile-ups, and loss of grain-boundary strength. They consider not only radiation-induced hardening and strain-localization effects, but also the grain-boundary changes due to the combined neutron irradiation and pressurized-water exposure up to 300 °C. The present SCC model focuses on, and is best adapted to, evaluating the initiation of intergranular cracks. The investigation of crack propagation requires an entirely different approach.

## 2. Materials and Methods

Our predictive intergranular crack initiation model is described in Section 2.1 and Section 2.2. Section 2.1 addresses dose-dependent modelling of plastic strain spreading at the grain scale, while Section 2.2 addresses the grain-boundary cohesive-energy evolution due to simultaneous neutron irradiation and reactor coolant exposure. The integrated model, combining the calculations from Section 2.1 and Section 2.2, is then tested (in Section 3) for an actual grade 300 steel case, as described in Section 2.3.

### 2.1. Grain Boundary Damage: Contribution of Plastic Strain Spreading Evolutions

Slip bands gradually form during the deformation spreading, as the applied load activates the dislocation sources initially present in the material. The formation of a new slip band requires that mobile dislocations overcome the critical cross-slip stress $\tau_{III}$ before generating a new secondary slip band; a new (primary) slip band is generated after secondary dislocations cross-slip back again into a shifted primary slip plane. The internal stress accumulation is due to the gradual formation of grain-wide dislocation pile-ups. In these conditions [5,6], slip bands are separated by characteristic spacing d:

(1) $$d=\left|\frac{D_{g}}{k_{max}}ln\left(\frac{\tau_{III}+\frac{1}{2}{S\cdot \sigma }_{app}}{{S\cdot \sigma }_{app}}\right)\right|$$

where $D_{g}$ is the effective grain size; $\tau_{III}$ is the (material-dependent) critical cross-slip stress; $k_{max}$ is a dimensionless scaling factor adjusting the pile-up stress decay rate normal to the primary slip plane, S is the (grain orientation-dependent) Schmid factor, $\sigma_{app}$ is the stress applied to the grain aggregate, accounting for the irradiation-hardening effect as per the disperse barrier model. This means $\sigma_{app}=\sigma_{YS}+M\alpha \mu b\sqrt{nD}$ where $\sigma_{YS}$ is the as-received material yield stress, n is the irradiation defect number density and D the irradiation defect size, M ≈ 3 is Taylor’s factor, α ≈ 0.3–0.4 is the dislocation/defect interaction strength coefficient, μ the material shear modulus, b is the Burgers vector modulus. Critical cross-slip stress $\tau_{III}$ refers to the Friedel-Escaig cross-slip model [19,20,21]; applicable to 300-type steels [22,23].

Slip band broadening is controlled by the accumulation of dislocations coming from secondary slip bands, traversing the band-to-band distance d between the primary slip bands. In these conditions [5,6], the characteristic slip band thickness e can be described as:

(2) $$e=\left|\frac{2d}{k_{max}}ln\left(\frac{\sigma_{defect}+\frac{1}{2}{S\cdot \sigma }_{app}}{S\cdot \sigma_{app}}\right)\right|$$

where d is the band spacing obtained from Equation (1); $\sigma_{defect}$ is the irradiation defect strength calculated based on the defect size using the Scattergood and Bacon expression [24]:

(3) $$\sigma_{defect}=\frac{\mu }{4\pi (1-\nu )}\frac{b}{L}\left[0.615-ln(\frac{b}{D}+\frac{b}{L})\right]$$

where ν is the Poisson coefficient, L is the average defect-to-defect distance. In practice, we use $L=1/\sqrt{nD}$ values best adapted to irradiated grain cases, i.e., austenitic grains including a mono-sized irradiation defect population [25,26]. The grain boundary stress due to plastic strain spreading can be estimated assuming that finite-thickness slip bands include mutually interacting dislocation pile-ups, positioned as sketched in Figure 1 below.

Stress $\tau_{gb}$ due to a given pile-up is evaluated at a specific calculation point (x,y) located in (or very near) the grain boundary, using:

(4) $$\tau_{gb}=-½\tau_{i}G\left(\xi \right)\left\{f\left(\xi ,\eta ,\varphi \right)\right\}$$

where $\tau_{i}$ is the shear stress level acting in the *i*-th pile-up, for applied stress levels $\sigma_{app}$ consistent with dose-dependent tensile strain $\varepsilon_{p}\approx \;0.2-0.5\%$; ξ = $2y_{i}/d$ where $y_{i}$ is the vertical distance to the calculation point (x,y) and d the slip band spacing associated with the *i*-th pile-up; η = $2x_{i}/D_{g}$ where $x_{i}$ is the horizontal distance to the calculation point (x,y); φ is the angular shift in point $\left(x_{i},y_{i}\right)$ with respect to point (x,y) and G (ξ), i.e., a polynomial fraction of ξ. Term f(ξ,η,φ) in Equation (4) is given in explicit form in references [25] and [27].

(5) $$G\left(\xi \right)=\sqrt[{4}]{{\left(\xi^{2}+0.1664\right)}^{2}+4\xi^{2}}/\left[\sqrt{4.3264+\xi^{2}}\sqrt{0.0064+\xi^{2}}\right]$$

The stress level acting on a specific grain-boundary location is a superimposition of the elastic stresses arising from all the active slip planes arranged as shown in Figure 1. The maximum stress level is then extracted (calculated) for each slip band and normalized to the yield stress of the initial (u-irradiated) material. This calculation yields a local, dimensionless stress concentration factor, where grain boundary damage susceptibility depends on a Crack susceptibility factor R, defined as:

(6) R=Crack susceptibility factor=angle factor×stress concentration factor

The angle factor term in Equation (6) depends on the misorientation angle between the slip direction of the highest Schmid factor and the tensile loading axis in the grain under consideration (cf. Figure 2 caption). The actual micro-crack initiation susceptibility factor also depends on the grain boundary cohesive energy changes, according to the local grain boundary cohesive energy evolutions ${∆\gamma }_{gb}$ [12]. The effect of this ${∆\gamma }_{gb}$ change on the irradiation embrittlement response of a polycrystal is evaluated as explained in the next section.

### 2.2. Grain Boundary Damage: Contribution of Grain Boundary Cohesive Energy Evolutions

Irradiation-assisted stress corrosion cracking (IASCC) depends on the stress amplitude achieved at grain boundaries during irradiation-induced strain localization. At elevated temperatures, specific solute elements (for example, Ni, Si, P…) initially present in the grain and the coolant are transported towards the grain boundaries, thereby affecting the local chemical composition and the corresponding grain boundary cohesive energy. Solute atom transport (diffusion) is significantly influenced by the irradiation defect cascades and concurrent interstitial and vacancy fluxes. For this reason, the RAS effect is generally distinct from other segregation mechanisms, both qualitatively (solute atom type) and quantitatively (concentration of specific solute atoms). The RAS effect can be characterized using high-resolution techniques (such as Auger spectroscopy, SIMS, EDX), focusing on a few selected grain boundaries. Consequently, modelling and characterizing all the complex mechanisms controlling the grain-boundary chemical composition is far beyond the scope of this paper. A simplified yet insightful approach is adopted instead, focusing on effective dose- and time-dependent evolutions of the grain-boundary cohesive energy.

The cohesive grain boundary energy is defined as the excess free energy associated with the presence of a grain boundary, with respect to a reference, unperturbed crystallographic lattice. This quantity physically depends on the atomic misfit across the interface under consideration. There is no simple method for determining the grain-boundary cohesive energy based on the crystallographic data alone for a given local chemical composition. We therefore utilize the simple Read-Shockley model [28], in which the grain boundaries are considered as a dense array of dislocations (as shown in [29], for example). In that case, the relation between the grain-to-grain disorientation angle Θ and the effective grain boundary energy change $∆\gamma_{gb}$ (in J/m^2^ units) is given by:

(7) $${∆\gamma }_{gb}=sin\left|\Theta \right|\{\frac{{∆U}_{core}}{b}-\frac{\mu b}{4\pi \left(1-\nu \right)}ln\left(sin\left|\Theta \right|\right)\}$$

where ${∆U}_{core}$ evolutions relate to the effective interfacial chemical composition evolutions due to irradiation and reactor coolant exposure. Equation (7) applies to random or vicinal grain boundaries [30], which are typically found in grade 300 steels. Other grain boundary types are also present but are neglected, for the sake of conservatism and simplicity. In principle, the quantity $∆U_{core}$ is also Θ-dependent according to the local variations in grain boundary chemical composition. Grain boundary energy changes ${∆\gamma }_{gb}$ also depend on the boundary plane orientation itself, which involves (up to four) additional degrees of freedom. Such information is, however, not directly accessible to standard (bi-dimensional) EBSD analysis. Establishing the precise $∆U_{core}$ evolutions for all grain boundary cases in a macroscopic polycrystal is generally beyond the usual experimental capacities [31,32,33,34,35].

In this paper, we simply assume that the grain boundary energy dispersion follows the variations in the misorientation angle Θ calculated as explained above. In other words, we assume that incorporating a large enough Θ distribution (see also Figure 2d) in Equation (7) yields a corresponding $∆\gamma_{gb}$ dispersion that is statistically representative of actual macroscopic grain aggregates. The impact of the $∆U_{core}$ evolution is evaluated through calculating the grain boundary energy evolutions under different, uniform imposed $∆U_{core}$ changes, proportional to a reference quantity $U_{core,0}$. In practice, the reference value $U_{core,0}$ can be determined using atomistic calculations, for undecorated dislocations in pure metallic systems. In the rest of this paper, we (arbitrarily) set $U_{core,0}$ ≈ 0.8 eV/Å or 1.25 × 10^−9^ J/m (see reference [36] for the r_D_ = 150 Å case).

In Section 3, we assume that large imposed ${∆U}_{core}$ evolutions are due to the transport of specific solute elements to and from the grain boundaries, negatively affecting the grain-boundary cohesive energy and corresponding crack-initiation susceptibility (phosphorous, for example). Conversely, small ${∆U}_{core}$ changes presumably correspond to cases where solute element migration is somewhat limited, yielding better grain boundary tolerance to radiation-induced evolutions. In grade 300 steels, the small ${∆U}_{core}$ case likely corresponds to the rapid growth of a protective oxide barrier, preventing or retarding any further evolution due to solute-element transport from the coolant to the grain-boundary inner interface. The $∆U_{core}$ range investigated (see Section 3.2) attempts to capture all transport-related evolutions in post-irradiated grain boundaries exposed to PWR coolant [18,37,38,39,40,41].

Changes in the grain boundary cohesive energy generally affect (reduce) the grain boundary tolerance to the applied load. This change can be evaluated by introducing an effective stress correction ${∆\tau }_{c}$, by analogy with brittle-material failure, which develops whenever the elastic energy stored in the (loaded) matrix exceeds the energy required to generate new surfaces, over a certain characteristic distance. We therefore assume that ${∆\gamma }_{gb}$ evolutions can be evaluated using Griffith’s relation [42]:

(8) $${∆\tau }_{c}=\sqrt{\frac{\pi }{1-\nu^{2}}\frac{µ{∆\gamma }_{gb}}{r}}$$

where $r=D_{g}$ is the characteristic fracture initiator size (The effective grain size $D_{g}$ is calculated as explained in Section 2.3.) and ${∆\gamma }_{gb}$ (in J/m^2^) is the local, dose-dependent change in the grain boundary cohesive energy, derived from Equation (7). Inserting the ${∆\tau }_{c}$ contribution (from Equation (8)) into the stress concentration factor (in Equation (6)) yields the same R level under a lower applied stress, within a given grain boundary. In other words, the inter-granular cracking susceptibility increases with ${∆\tau }_{c}$ and, hence, with the grain boundary energy change ${∆\gamma }_{gb}$. In practice, the quantity ${∆\tau }_{c}$ is calculated for different ${∆\gamma }_{gb}$ levels according to systematic ${∆U}_{core}$ variations that depend on the local grain-to-grain misorientation angle Θ (see also Section 3.1). A comparison between the R factor evolutions and experimental post-irradiation data (the dose-dependent, non-cracking stress level, for example) may help identify the ${∆U}_{core}$ range consistent with the actual radiation response of the material.

### 2.3. Material Characterization and Related Input Data Extraction

The integrated crack initiation model of Section 2.1 and Section 2.2 shall be evaluated using the actual EBSD input dataset case as shown in Figure 2. The selected series 300 steel specimen was mechanically polished down to ¼ mm diamond paste, followed by vibratory polishing in OPS solution for 10 h. EBSD was then performed using a JEOL JSM 7001F Field-Emission SEM (JEOL, Tokyo, Japan) operated at 30 kV. All the details regarding the fabrication and chemical composition of the present specimen are available elsewhere [43]. The crystallographic orientation (Euler angles) of each electron backscattering diffraction (EBSD) grid point (taken as a sub-region of the grain aggregate). In practice, the orientation of each pixel is given by the matrix G defined as:

(9) $$G=\left[\begin{array}{ccc} cos\phi_{1}cos\phi_{2}-cos\theta sin\phi_{1}sin\phi_{2} & sin\phi_{1}cos\phi_{2}+cos\theta cos\phi_{1}sin\phi_{2} & sin\theta sin\phi_{2}\\ -cos\phi_{1}sin\phi_{2}-cos\theta sin\phi_{1}cos\phi_{2} & -sin\phi_{1}sin\phi_{2}+cos\theta cos\phi_{1}cos\phi_{2} & sin\theta cos\phi_{2}\\ sin\theta sin\phi_{1} & -sin\theta cos\phi_{1} & cos\theta \end{array}\right]$$

where $\phi_{1}$, θ and $\phi_{2}$ are Euler angles with Bunge convention [44]. A grain-dependent vector $r_{1}$ expressed in the microscopic coordinate system (crystallographic direction $r_{1}$ = [111] for example) can be expressed in the grain-independent macroscopic system as vector $r_{1}^{\prime }$ using Equation (10):

(10) $$r_{1}^{\prime }=G\cdot r_{1}$$

The effective grain size assigned to each pixel is then: $D_{g}^{eff}=M_{\theta }\delta$ where $M_{\theta }$ is the number of consecutive measurements yielding Θ ≤ 5° along the loading direction (parallel to the horizontal axis) and δ is the measurement step. It is assumed that $D_{g}^{eff}$ is a fair approximation of the (maximal) gliding distance of dislocation lines sitting within a given grain, at or near the considered calculation point. In the rest of the paper, any reference to grain size relates to $D_{g}^{eff}$ unless otherwise specified. A specific vector $r_{1}^{\prime }$ is first assigned to each grid point and then used to evaluate the angles $\Theta_{H}$ and $\Theta_{V}$ characterizing the adjacent vertical and horizontal pixel disorientation, everywhere in the grain aggregate. The disorientation angle Θ assigned to a given pixel is simply the maximum of $\Theta_{H}$ and $\Theta_{V}$. We further verified that using different Θ calculation approaches, like using different reference vectors $r_{1}$ or putting $\Theta =\Theta_{H}$ (for example) yields qualitatively the same trends as shown in Figure 2b.

Our model predictions shall be finally compared with Slow Strain Rate Tensile Test results (see Section 3 below), where grade 300 steel specimens (cf. Figure 2) are exposed to PWR «primary water», which typically contains 25–35 cc/kg H2 STP, 1000 ppm B, 2 ppm Li [43].

## 3. Results

### 3.1. Grain Boundary Cracking Susceptibility Evaluation: Assumptions and Input Data Processing

The calculation of Damage factor R associated with Figure 2 input data is carried out as explained in Figure 3, where we also assume:

1. the tensile loading axis is horizontal (cf. Figure 2a); however, we checked that this condition has little or no effect on the post-irradiation trends, provided the investigated grain aggregate includes at least 100 grains.
2. the applied stress level is uniform throughout the grain aggregate, though the internal stress is not;
3. plastic strain develops in single slip conditions, in consistency with direct observations of post-irradiated materials (see, for example, [45]),
4. the mutual, grain-to-grain interactions are neglected: separate investigations confirm this is a reasonable approximation [46,47], especially in the Figure 2b case study (monomodal, equiaxed grain size distribution).

**Figure 3:**
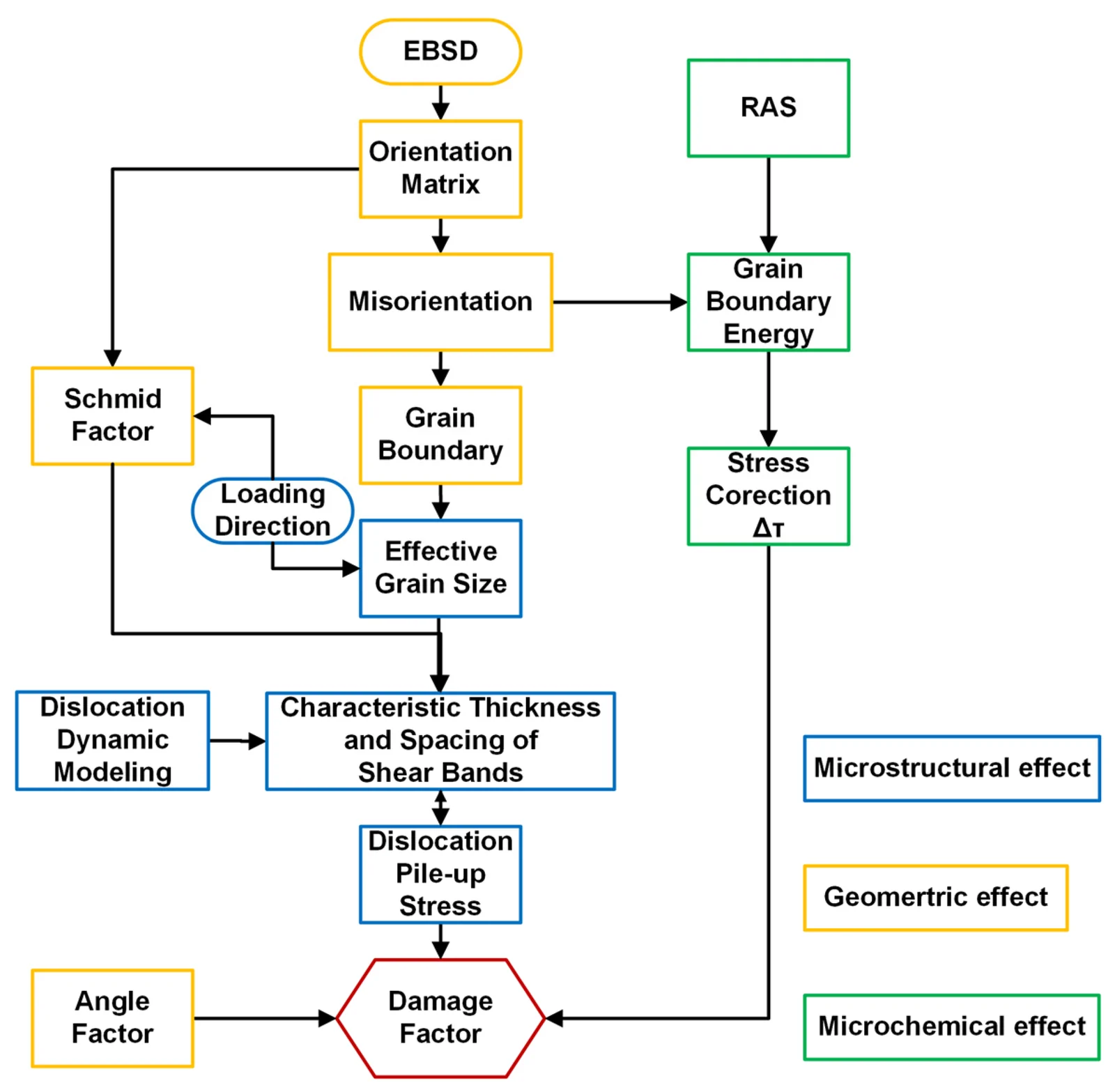
Damage factor «*R*» calculation procedure for different loading, irradiation and reactor coolant exposure conditions. Factor «*R*» distribution directly relates to crack-initiation susceptibility as applicable to a macroscopic, representative polycrystalline grain aggregate.

The utilization of assumptions 2, 3, and 4 entails the following comments. Nonuniform applied stress conditions are typical of post-necking strain levels, which occur far beyond the micro-crack initiation stage. Addressing non-uniform applied stress may help analyze micro-crack initiation in notched or sub-sized specimens, which is, however, beyond the scope of this paper. In post-irradiated strained specimens, micro-crack initiation usually takes place where (single) slip in one grain is not transmitted to the adjacent one, thereby producing a high stress concentration. Multiple slip, on the other hand, acts as a local stress mitigation mechanism and, for this reason, is generally unrelated to micro-crack initiation. In many cases, multiple slip activation in one particular grain is incidentally due to grain-to-grain interaction itself, at least up to several percent plastic strain. Assumptions 3 and 4 are therefore especially well adapted to the context of the present investigation.

The slip band thickness and spacing results hence vary from one EBSD pixel to another, in order to satisfy conditions 1–4 everywhere in the aggregate. In practice, Equations (1)–(8) are evaluated using actual EBSD grain size and orientation data cases as shown in Figure 2, collected from the model grain aggregate comprising 1124 grains. The spacing between each measurement line is set to 2.92 µm, covering the whole grain aggregate as shown in Figure 2a.

### 3.2. Grain Boundary Cracking Susceptibility Evaluation: Main Trends and Results

The effect of the imposed ${∆U}_{core}$ amplitude is evaluated as shown in Figure 4, for fixed loading level and irradiation conditions (see the accompanying caption). Applying an increasing uniform ${∆U}_{core}$ amplitude yields a higher average damage factor and, notably, a broader *R* factor distribution, for a given applied stress amplitude. If inter-granular crack initiation corresponds to a fixed damage factor threshold *R*th. This means that the grain boundary tolerance to the applied load decreases with increasing ${∆U}_{core}$ and the corresponding ${∆\tau }_{c}$ amplitude. In other words, the Figure 3 procedure returns a larger count satisfying *R* ≥ *R*th with increasing ${∆\tau }_{c}$ amplitude.

It is generally understood that grain boundaries presenting a relatively large grain-to-grain slip contrast generally correspond to a high intergranular crack initiation probability [48,49,50,51,52]. Plotting the damage factor results in the form of damage factor maps (not shown) may then help evaluate the number density of high crack-initiation-susceptibility sites within the grain aggregate [25]. It is, however, important to note that slip transfer at slip-band/grain-boundary intersections cannot be fully characterized by 2D EBSD observations, as they miss grain-depth information (see Section 3.1).

Damage factor distributions associated with the Figure 2 case study are shown in Figure 5, for several applied stress levels, irradiation doses, and cohesive grain boundary energy evolutions. In these conditions, it can be shown that slip band thickness and spacing are consistent with corresponding high-resolution SEM and AFM observations [25], up to at least several percent strain level [53]. However, the occurrence (count number) of the smallest slip band thickness (<50 nm) and spacing (<500 nm) is often overpredicted (by up to 50%). Such deviations have virtually no impact on the R > 5 count numbers, however.

Interestingly, we noted that failed grain boundaries can generally be associated with *R*th > 5 (cf. Figure 5 and Figure 6 and accompanying captions). In practice, we also noted that *R*th ~ 5 corresponds to about 1 GPa in grade 300 steels; an applied stress level associated with tensile specimen failure and ε_p_ ≈ 0 after high-dose irradiation (In case a specimen fails for ε_p_ ≈ 0 it follows that: max. tensile stress ≈ grain boundary failure stress) [2,3,4,5]. The threshold *R*th should generally depend on the material considered and exposure conditions. At least empirically, however, *R*th ~ 5 applies to significantly different material chemical compositions, yield stresses, reactor coolants, and crystallographic microstructures [53,54].

The investigated 0.1 dpa dose level corresponds to an irradiation defect number density *n* = 4 × 10^21^ m^−3^; the 0.5 dpa dose level to 4 × 10^22^ m^−3^; and the 3 dpa dose level to *n* = 4 × 10^23^ m^−3^, assuming a fixed irradiation defect size of D = 5 nm. The predicted irradiation response (R factor evolutions) generally depends on the *n*D product associated with a particular dose and irradiation temperature, as pointed out in earlier studies [55,56]. That specific trend holds true whenever *n*D ∝ $\sigma_{app}$, as defined in Section 2.1. This applies to various metallic materials, corresponding grain microstructures, and various neutron doses and temperatures, in the presence of irradiation defect sizes up to D = 20 nm and number densities up to *n* = 4 × 10^24^ m^−3^ [54].

Furthermore, the average dose-dependent evolution of the R factor usually depends on the reciprocal of the initial material strength. High-strength materials habitually include reinforcing particles that provide a larger irradiation-defect-sink capacity and, hence, better radiation tolerance. Factor R distribution width scales with the grain size distribution amplitude. A monomodal, narrow grain-scale distribution presents smaller maximal R values and hence, a lower susceptibility to intergranular crack initiation.

Our model is applicable to grain sizes ranging between $D_{g}$ = 1 µm and $D_{g}$ = 100 µm. A lower $D_{g}$ limit actually exists that allows slip band development as per the plasticity mechanisms described in Section 2.1. Below this limit, grain boundary sliding competes with slip band development in smaller grains. Conversely, an upper $D_{g}$ limit also exists where grain boundary pile-ups compete with intragranular pile-ups due to dislocation locks (for example).

Grain orientation and corresponding Schmid factor dispersions are minimal in grade 300 steels exhibiting an equiaxed grain microstructure. Conversely, these dispersions are maximal in highly textured, drawn materials [57]. Slip band thickness and spacing distributions are correctly captured in both cases; nevertheless, this is provided the calculations (assuming Section 2.1 plasticity mechanisms) include the proper EBSD data input.

The impact of both the large and small ${∆U}_{core}$ cases is evaluated, for the sake of comparison and understanding. Large, imposed ${∆U}_{core}$ variations (e.g., ${∆U}_{core}$ = 10 $U_{core,0}$) represent significant grain-boundary composition evolutions, consistent with the absence of a protective oxide scale, at the grain-boundary interface with the coolant. Conversely small ${∆U}_{core}$ variations (e.g., ${∆U}_{core}$ ≤ $U_{core,0}$) represent limited grain boundary evolutions, consistent with the presence of an oxide barrier there. Figure 5 results indicate an applied intergranular crack initiation stress of about 700 MPa at 3 dpa using a uniform evolution amplitude ${∆U}_{core}$= $U_{core,0}$. This prediction is consistent with corresponding experimental observations and analysis [58,59,60,61].

The results in Figure 6 are obtained after normalization of the applied stress level with respect to the material yield stress, which depends on the dose level. Such a comparison allows the evaluation of the grain aggregate tolerance to the applied load amplitude, depending on the exposure conditions under consideration. At an irradiation dose of 0.5 dpa and ${∆U}_{core}=10U_{core,0}$ for instance, no-failure conditions correspond to an applied load as low as 0.36 times the yield stress (Figure 6b and caption), whereas the (average) failure stress level is 1.09 times the yield stress (Figure 6a and caption).

Direct comparison with corresponding experimental (slow strain rate tensile tests: «SSRTT») results [59,60,61] and confirms the good representativity of the imposed ${∆U}_{core}$ range (e.g., from 0.1 to 10 times $U_{core,0}$) for the material and exposure cases investigated. The failure-stress versus no-failure stress gap, however, decreases with increasing dose, denoting a significant dose effect on the grain boundary stress distributions, for a given ${∆U}_{core}$ case (cf. Figure 5).

**Figure 6:**
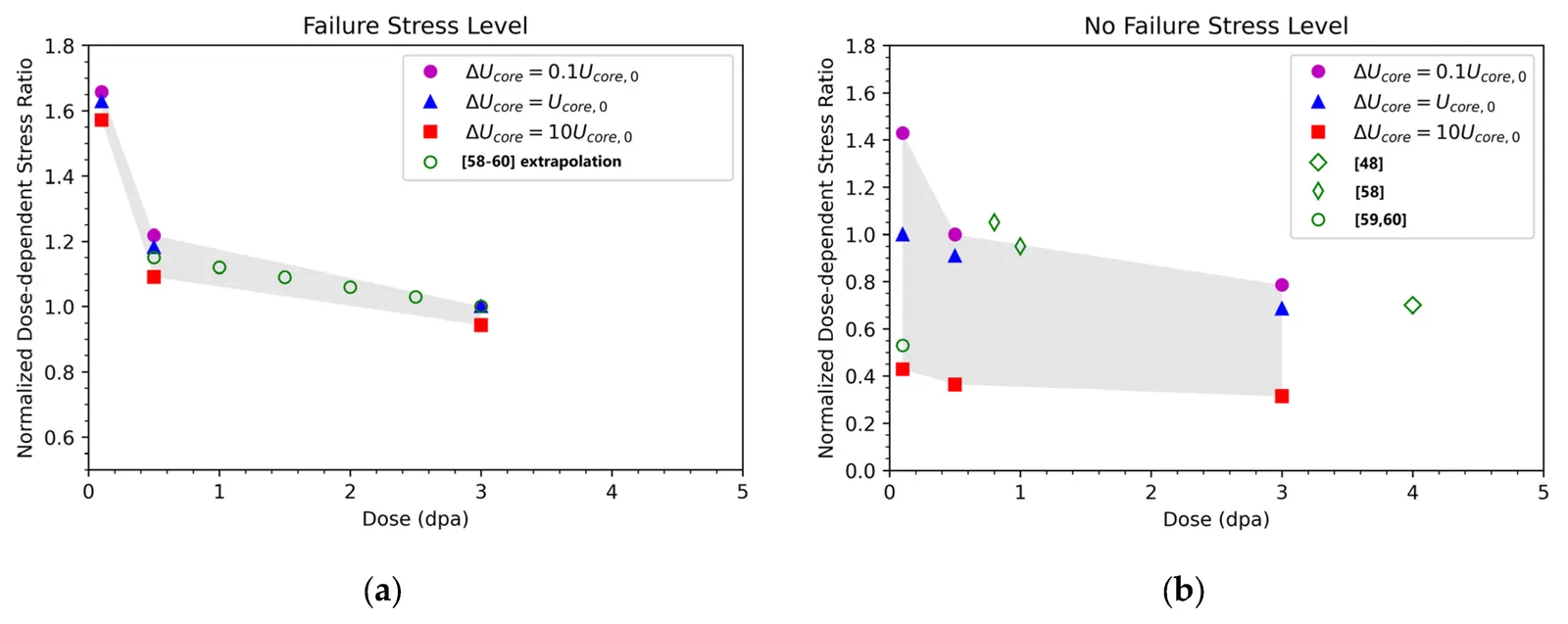
Dose-dependent tolerance to applied stress of grade 300 steel irradiated at 300 °C. (**a**) The average failure stress level evolution with dose corresponds to a 50% probability of achieving *R* = 5 throughout the grain aggregate. (**b**) The no-failure stress level obtained is associated with a 90% probability of obtaining *R* ≤ 5 across the grain aggregate. Comparative experimental (SSRTT) data are extracted from [48,58,59,60] as shown. The gray shaded area represents the modeled scattering due to the combined effects of plasticity and grain boundary strength.

Overall, ${∆U}_{core}$ changes smaller than $0.1U_{core,0}$ are applicable to dose cases much lower than 0.5 dpa [62]. On the other hand, ${∆U}_{core}$ changes larger than $10U_{core,0}$ generally produce unrealistically large RAS-driven evolutions, in the context of PWR utilization.

As a concluding remark, the present radiation embrittlement model demonstrates promising agreement with available experimental trends and can be extended to a much broader range of cases, following further development and validation. Obtaining detailed ${∆U}_{core}$ expressions [63,64] would, for example, help address different crack initiation mechanisms in various materials, exposure conditions (dose, temperature) and reactor coolants (liquid metal, molten salts, gas). Addressing the very high-dose irradiation domain (Beyond 10 dpa: in case of long-term PWR operation or in fast reactor conditions) is also possible, provided that specific high-dose hardening mechanisms and their related ${∆U}_{core}$ evolutions are investigated first.

Our approach was initially applied for the comparative ranking of materials, as presented in various research projects. In the future, it can also be used for assessing reactor internals lifetime, if combined with appropriate in situ experimental support. For instance, a practical component lifetime evolution method is currently under development based on Section 2 arguments combined with micro-indentation, temperature and irradiation dose monitoring, in PWR and other advanced reactor types.

## 4. Conclusions

This paper presents a comprehensive evaluation method, aiming to capture influential IASCC mechanisms in grade 300 steels. Grain boundary crack initiation susceptibility and its dose-dependent evolution are analyzed across a broad range of PWR operational conditions, accounting for various relevant variables (high temperature, irradiation dose, mechanical load, grain size, grain orientation, grain boundary configuration, etc.).

The irradiation defect dispersions affect the related slip band spreading characteristics, in terms of slip band thickness and spacing. The corresponding grain boundary stress and its dose-dependent evolution are evaluated in each case. The concurrent grain-boundary cohesive energy evolution also contributes to intergranular crack-initiation susceptibility. These changes are accounted for using a simplified approach based on ${∆U}_{core}$ quantity, from the Read-Shockley grain boundary model. The above implemented effects are evaluated and rationalized in terms of the evolutions of the dimensionless factor «*R*».

In practice, the predicted R factor evolutions relate to the initial material strength and to the *n*D product associated with a particular dose and irradiation temperature (recalling D is the irradiation defect size and *n* the irradiation defect density). The R-factor distribution broadness scales with the grain size distribution width: a narrow grain size distribution presents smaller maximal R factor amplitude and hence, a lower susceptibility to intergranular crack initiation.

Our model is applicable to grain sizes ranging between $D_{g}$ = 1 µm and $D_{g}$ = 100 µm, in relation to the considered plasticity mechanisms. The grain size and orientation distributions can be correctly captured using applicable EBSD map input, as prescribed in Section 2.

The model predictions are in good agreement with the corresponding SSRTT measurements for grade 300 steels. For instance, imposing ${∆U}_{core}$ = $U_{core,0}$ evolutions is consistent with grain boundary crack initiation for an applied stress level of 700 MPa, after exposure to neutron irradiation up to 3 dpa at 300 °C in primary water. Following further development, this approach may be extended to other materials, irradiation conditions and utilized for assessing the reactor internals lifetime.

## Figures and Tables

**Figure 1:**
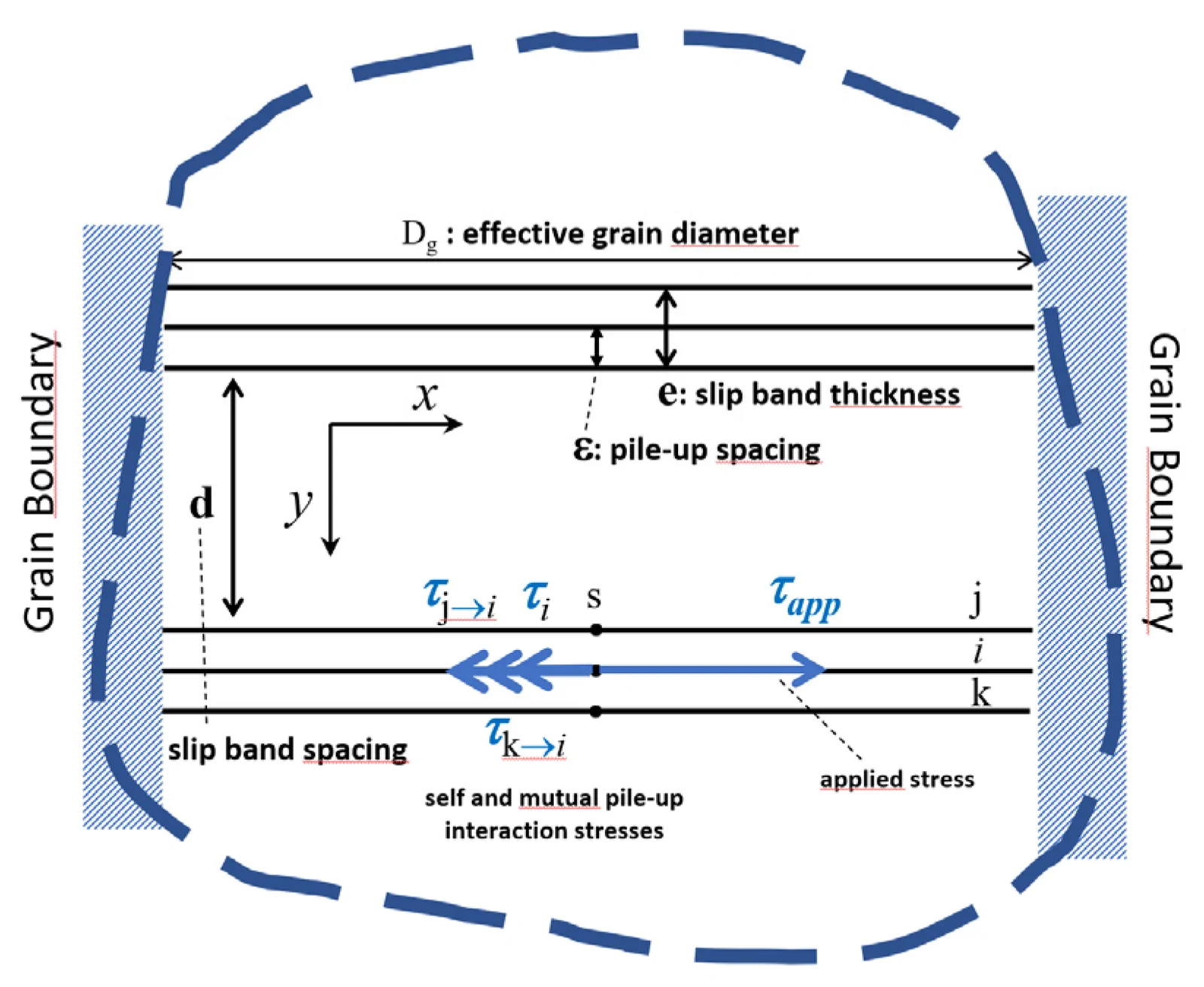
Schematic of plastic strain spreading in a grain of size $D_{g}$: a set of *N* pile-ups emitted from dislocation source «s». The slip bands are spaced a minimum distance d apart. The thickness of an individual slip band corresponds to the value *e*, where the sub-slip band pile-ups are separated by a uniform distance ε for simplicity. Each individual slip band includes three distinct dislocation pile-ups labelled *i*, j and k as shown. The stress acting on pile *i* is the superimposition of the applied stress $\tau_{app}$, the self-stress $\tau_{i}$ and the mutual interaction stresses $\tau_{j\to i}$ and $\tau_{k\to i}$ due to pile-ups j and k, respectively (blue arrows). It can be shown that the stress due to the adjacent slip bands, sitting at a minimal distance «d» from slip band «*i*, j, k», is negligibly small. The wavy red zones highlight the effective grain boundary locations associated with the slip bands shown in this figure.

**Figure 2:**
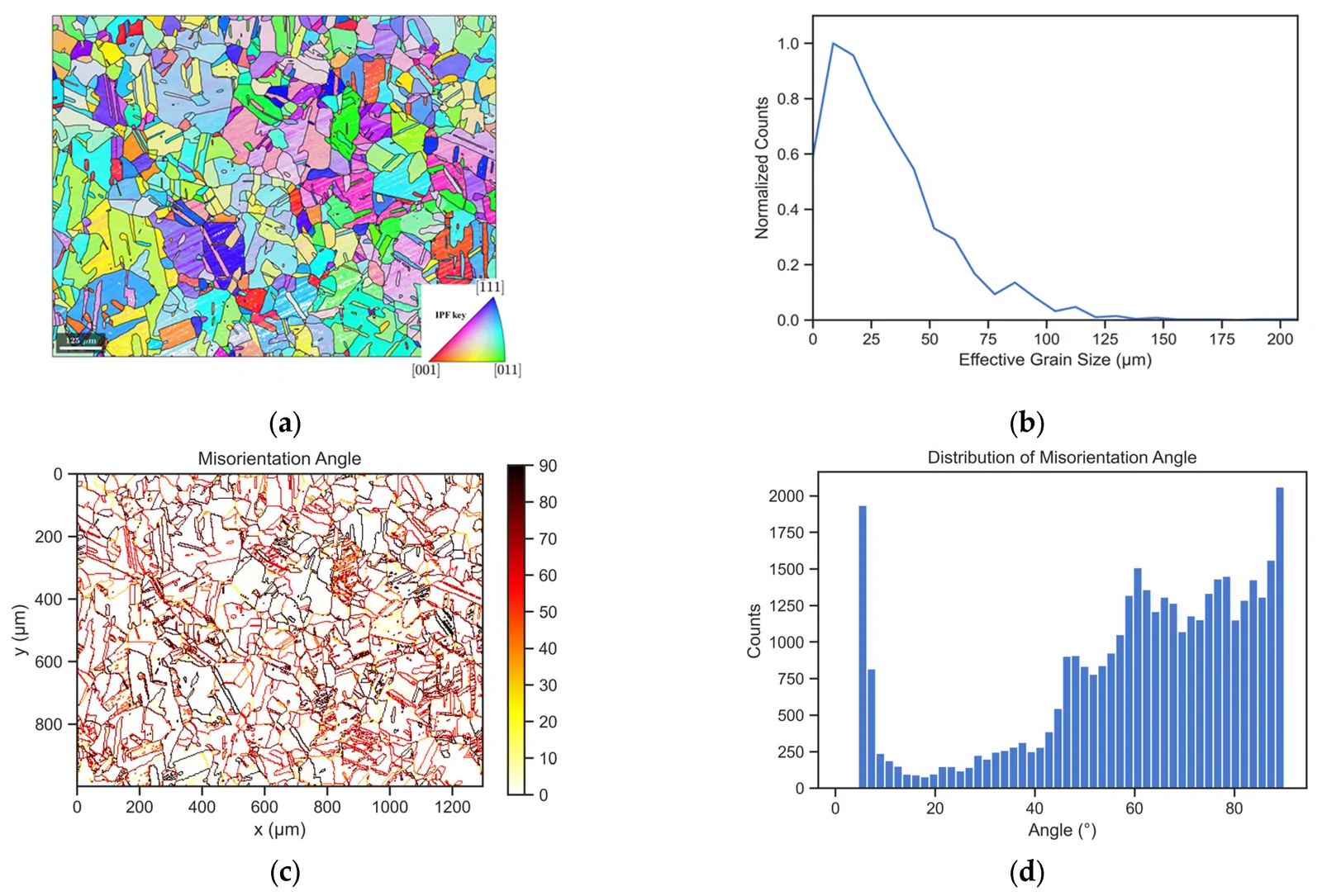
Representative grain aggregate data used for grain boundary stress calculation in grade 300 steels. (**a**) EBSD inverse pole figure (IPF) map, scale bar = 125 µm. (**b**) Corresponding effective grain size distribution. The horizontal EBSD scan direction aligns with the tensile loading direction applied in the Section 3.1 calculation procedure. (**c**) Grain-to-grain misorientation angle Θ map and (**d**) corresponding misorientation angle Θ distribution. The crystallographic direction [111] is used as the reference crystallographic direction.

**Figure 4:**
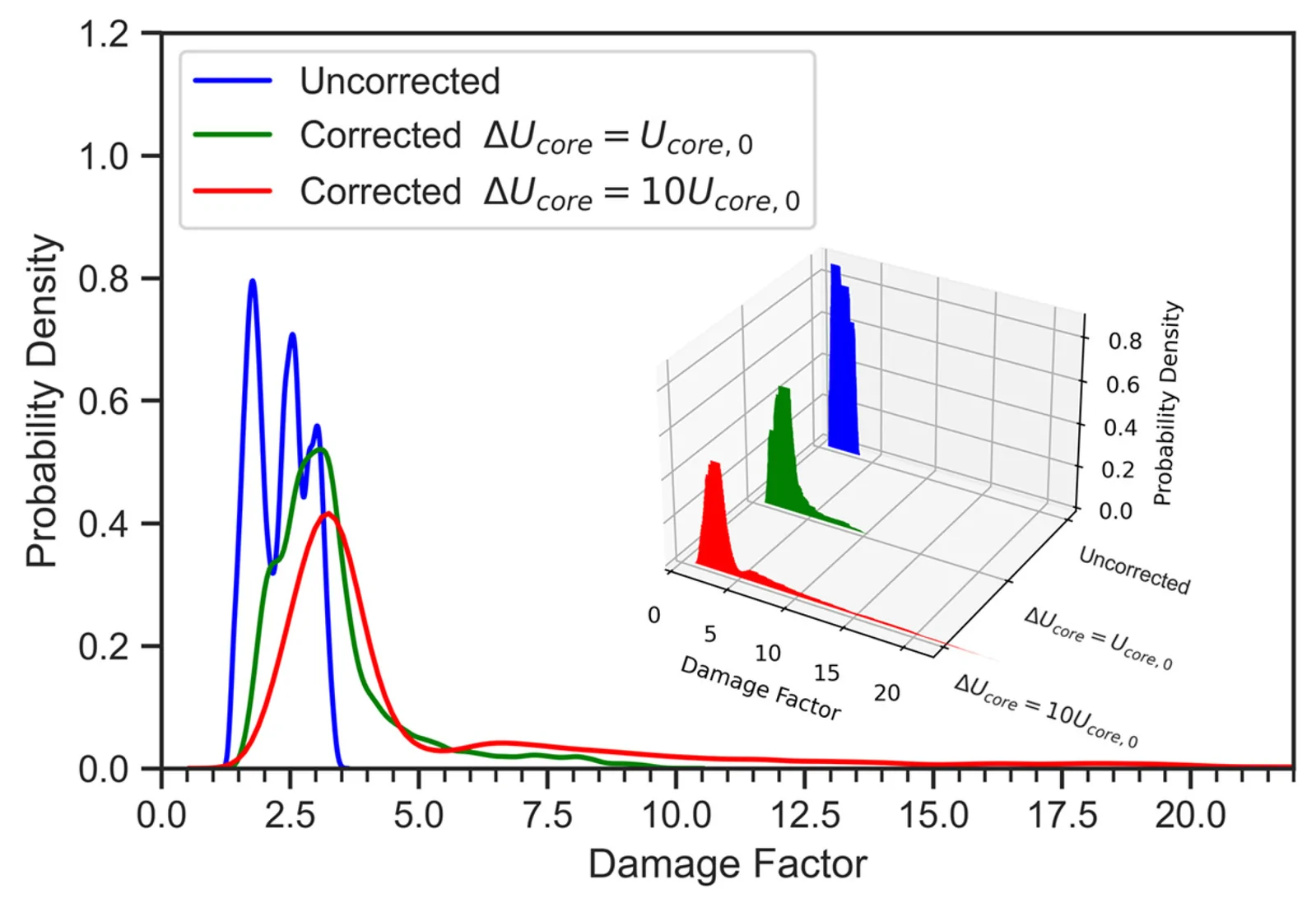
Effect of ${∆U}_{core}$ evolution on the damage factor «*R*» distribution. The results shown correspond to an irradiation defect density of 4 × 10^22^ m^−3^, a defect size of 5 nm and an applied stress of 375 MPa. The irradiation conditions considered correspond to 0.5 dpa at 300 °C.

**Figure 5:**
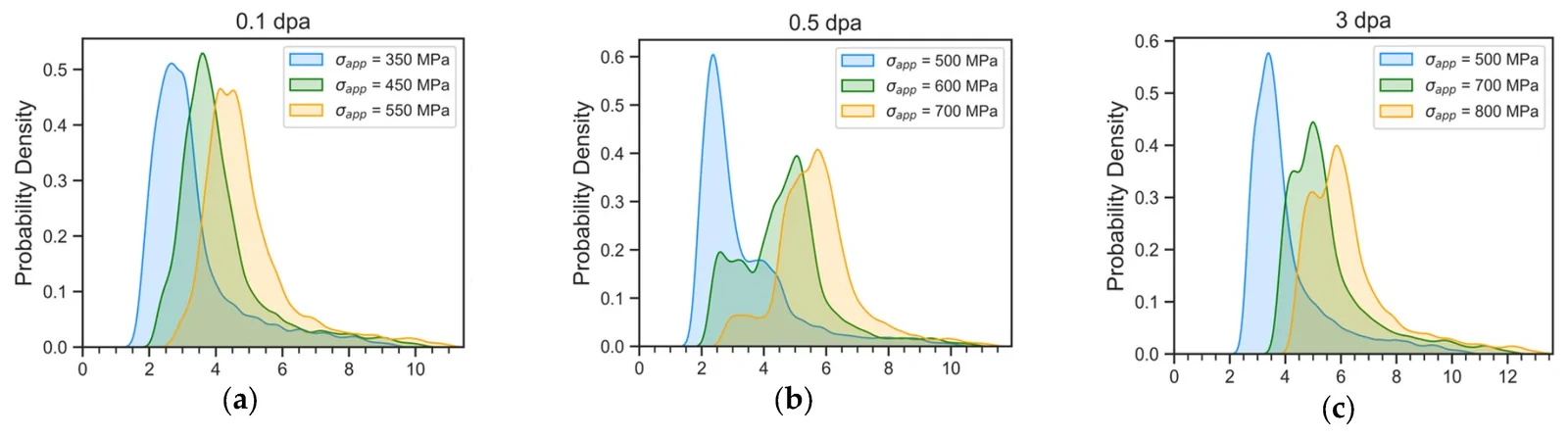

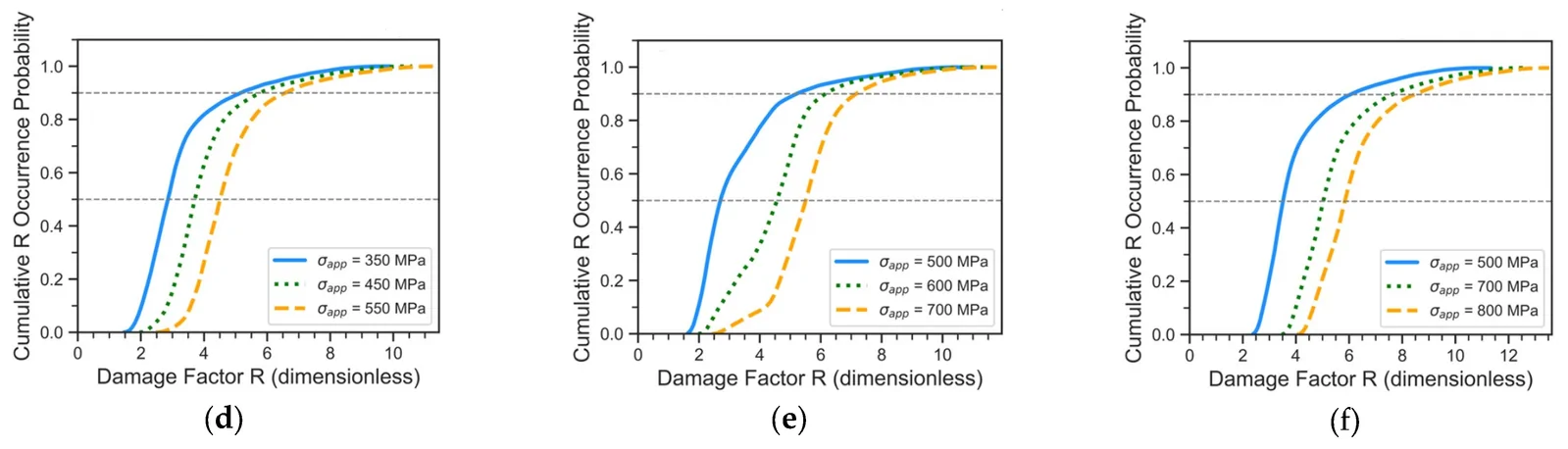
Effect of loading and irradiation dose conditions on the damage factor «*R*» distribution using ${∆U}_{core}=U_{core,0}$. Probability density distribution and cumulative probability distribution of the damage factor «*R*» following irradiation doses: 0.1 dpa (**a**,**d**); 0.5 dpa (**b**,**e**) and 3 dpa (**c**,**f**).

## Data Availability

The original contributions presented in this study are included in the article. Further inquiries can be directed to the corresponding author.

## Abbreviations

The following abbreviations are used in this manuscript:

PWR	Pressurized Water Reactors
EBSD	Electron Backscatter Diffraction
IASCC	Irradiation Assisted Stress Corrosion Cracking
GB	Grain Boundary
RAS	Radiation-Assisted Segregation
SSRTT	Slow Strain Rate Tensile Tests

## References

[1] Pokor C., Bréchet Y. (2004). Dubuisson, Irradiation damage in 304 and 316 stainless steels: Experimental investigation and modelling. Part I: Evolution of the microstructure. J. Nucl. Mat..

[2] Bruemmer S.M., Simonen E.P., Scott P.M., Andresen P.L., Was G.S., Nelson J.L. (1999). Radiation-induced material changes and susceptibility to intergranular failure of light-water-reactor core internals. J. Nucl. Mat..

[3] Murtrey M., Cui B., Robertson I., Farkas D., Was G. (2015). Mechanism of dislocation channel-induced irradiation assisted stress corrosion crack initiation in austenitic stainless steel. Curr. Opin. Solid State Mat. Sci..

[4] Jiao Z., Was G.S. (2008). Localized deformation and IASCC initiation in austenitic stainless steels. J. Nucl. Mat..

[5] Gururaj K., Robertson C., Fivel M. (2015). Channel formation and multiplication in irradiated FCC metals: A 3D dislocation dynamics investigation. Philos. Mag. A.

[6] Gururaj K., Robertson C., Fivel M. (2015). Post-irradiation plastic deformation in bcc Fe grains investigated by means of 3D dislocation dynamics simulations. J. Nucl. Mat..

[7] Arsenlis A., Rhee M., Hommes G., Cook R., Marian J. (2012). A dislocation dynamics study of the transition from homogeneous to heterogeneous deformation in irradiated body-centered cubic iron. Acta Mater..

[8] Jiao Z., Was G.S. (2011). Impact of localized deformation on IASCC in austenitic stainless steels. J. Nucl. Mat..

[9] Tucker R.P., Wechsler M.S., Ohr S.M. (1969). Dislocation Channeling in Neutron-Irradiated Niobium. J. Appl. Phys..

[10] GWas S., Alexandreanu B., Busby J. (2004). Localized deformation induced IGSCC and IASCC of austenitic alloys in high temperature water. Key Eng. Mater..

[11] Renault-Laborne A., Hure J., Malaplate J., Gavoille P., Sefta F., Tanguy B. (2018). Tensile properties and deformation microstructure of highly neutron-irradiated 316 stainless steels at low and fast strain rate. J. Nucl. Mater..

[12] Karlsen W., Diego G., Devrient B. (2010). Localized deformation as a key precursor to initiation of intergranular stress corrosion cracking of austenitic stainless steels employed in nuclear power plants. J. Nucl. Mater..

[13] Monnet G., Mai C. (2019). Prediction of irradiation hardening in austenitic stainless steels: Analytical and crystal plasticity studies. J. Nucl. Mater..

[14] Berveiller M., Adjanor G., Latourte F., Vidal J., Gosset A. Presentation of the SOTERIA Numerical Platform for the Assessment of Ageing Mechanisms in RPV and Internals. Proceedings of the International Conference on Nuclear Power Plant Life Management.

[15] Hure J., El Shawish S., Cizelj L., Tanguy B. (2016). Tanguy, Intergranular stress distributions in polycrystalline aggregates of irradiated stainless steel. J. Nucl. Mater..

[16] Scherer J.M., Besson J., Forest S., Hure J., Tanguy B. (2019). Strain gradient crystal plasticity with evolving length scale: Application to voided irradiated materials. Eur. J. Mech. A Solids.

[17] Marano A., Gélébart L., Forest S. (2019). Intra-granular localization induced by softening crystal plasticity: Analysis of slip and kink bands localization modes from high resolution FFT-simulations results. Acta Mater..

[18] He M.-G., Johnson D.C., Was G.S., Robertson I.M. (2017). The role of grain boundary microchemistry in irradiation-assisted stress corrosion cracking of a Fe-13Cr-15Ni alloy. Acta Mater..

[19] Puschl W. (2002). Models for dislocation cross-slip in close-packed crystal structures: A critical review. Prog. Mater. Sci..

[20] Kelly A.A., Knowles K.M. (2012). Crystallography and Crystal Defects.

[21] Escaig B. (1968). Cross slip of dislocations in the face centered cubic structure. J. Phys..

[22] Robertson C.F., Fivel M.C., Fissolo A. (2001). Dislocation substructure in 316L stainless steel under thermal fatigue up to 650 K. Mat. Sci. Eng. A.

[23] Robertson C., Li Y., Marini B. (2022). Dose-dependent strain localization and embrittlement in ferritic materials: A predictive approach based on sub-grain plasticity modelling. J. Nucl. Mater..

[24] Bacon D.J., Kocks U.F., Scattergood R.O. (1973). The effect of dislocation self-interaction on the Orowan stress. Philos. Mag..

[25] Wang Y., Zitao Z., Robertson C. (2003). Prediction of inter-granular crack initiation susceptibility in post-irradiated grade 300 steels. Mod. Sim. Mat. Sci. Eng..

[26] Malerba L., Caturla M.J., Gaganidze E., Kaden C., Konstantinović M.J., Olsson P., Robertson C., Rodney D., Ruiz-Moreno A.M., Serrano M. (2021). Multiscale modelling for fusion and fission materials: The M4F project. J. Nucl. Ener..

[27] Hirth J.P., Lothe J. (1992). Theory of Dislocations.

[28] Shockley W., Read W.T. (1949). Quantitative Predictions from Dislocation Models of Crystal Grain Boundaries. Phys. Rev..

[29] Liu B., Eisenlohr P., Roters F., Raabe D. (2012). Simulation of dislocation penetration through a general low-angle grain boundary. Acta Mater..

[30] Gjostein N.A., Rhines F.N. (1959). Absolute interfacial energies of [001] tilt and twist boundaries in copper. Acta Met..

[31] Jokl M.L., Vitek V., McMahon C.J. (1980). A microscopic theory of brittle fracture in deformable solids: A relation between ideal work to fracture and plastic work. Acta Met..

[32] Ortner S.R., Hippsley C.A. (1996). Two component description of ductile to brittle transition in ferritic steel. Mater. Sci. Technol..

[33] Tweed J.H., Knott J.F. (1987). Micromechanisms of failure in CMn weld metals. Acta Met..

[34] Veistinen M.K., Lindroos V.K. (1984). Evaluation of the effective surface energy of ferrite in a 26Cr-1Mo ferritic stainless steels. Scr. Met..

[35] McMahon C.J., Vitek V. (1979). The effects of segregated impurities on intergranular fracture energy. Acta Met..

[36] Aslanides A., Pontikis V. (1998). Atomistic study of dislocation cores in aluminium and copper. Comp. Mat. Sci..

[37] Cui B., McMurtrey M.D., Was G.S., Robertson I.M. (2014). Micromechanistic origin of irradiation-assisted stress corrosion cracking. Philos. Mag..

[38] Paccou E., Tanguy B., Legros M. (2020). Irradiation-assisted stress corrosion cracking susceptibility and mechanical properties related to irradiation-induced microstructures of 304L austenitic stainless steel. J. Nucl. Mater..

[39] Gussev M.N., Leonard K.J. (2019). In situ SEM-EBSD analysis of plastic deformation mechanisms in neutron-irradiated austenitic steel. J. Nucl. Mater..

[40] Johnson D.C., Kuhr B., Farkas D., Was G.S. (2016). Quantitative analysis of localized stresses in irradiated stainless steels using high resolution electron backscatter diffraction and molecular dynamics modeling. Scr. Mater..

[41] Was G.S., Busby J.T. (2005). Role of irradiated microstructure and microchemistry in irradiation-assisted stress corrosion cracking. Philos. Mag..

[42] Griffith A.A. (1921). The phenomena of rupture and flow in solids. Phil. Trans. R. Soc. Lond..

[43] Gupta J., Hure J., Tanguy B., Laffont L., Lafont M.-C., Andrieu E. (2016). Evaluation of stress corrosion cracking of irradiated 304L stainless steel in PWR environment using heavy ion irradiation. J. Nucl. Mater..

[44] (1982). Texture Analysis in Materials Science: Mathematical Methods.

[45] Was G., Busby J., Jiao Z. (2006). The Use of Proton Irradiation to Determine IASCC Mechanisms in Light Water Reactors—Phase 3: Deformation Studies.

[46] Sauzay M., Osterstock S., Robertson C., Degallaix S., Aubin V., Hutar P. Influence de l’élasticité et de la plasticité cristallines sur la distribution des champs mécaniques à la surface des polycristaux. Proceedings of the CFM2007, 18ème Congrès Français de Mécanique.

[47] Déprés C., Reddy G.V.P., Robertson C., Fivel M. (2014). An extensive 3D dislocation dynamics investigation of stage-I fatigue crack propagation. Philos. Mag. A.

[48] Shen Z., Wagoner R.H., Clark W.A.T. (1986). Dislocation pile-up and grain boundary interactions in 304 stainless steel. Scr. Met..

[49] Nishioka H., Fukuya K., Fujji K., Kitsunai Y. (2008). Deformation structure in highly irradiated stainless steels. J. Nucl. Sci. Technol..

[50] Smith L., Farkas D. (2014). Non-planar grain boundary structures in fcc metals and their role in nanoscale deformation mechanisms. Philos. Mag..

[51] Schramm R.E., Reed R.P. (1975). Stacking fault energies of seven commercial austenitic stainless steels. Met. Trans. A.

[52] Rhodes C.G., Thompson A.W. (1977). The composition dependence of stacking fault energy in austenitic stainless steels. Met. Trans. A.

[53] Robertson C., Dupuy L., Hernandez-Mayoral M. Dislocation dynamics: Rationalisation of shear band formation through micro-models. Proceedings of the M4F European project Final Workshop.

[54] Bergner F., Hernández-Mayoral M., Heintze C., Konstantinovic M.J., Malerba L., Pareige C. (2020). TEM Observation of Loops Decorating Dislocations and Resulting Source Hardening of Neutron-Irradiated Fe-Cr Alloys. Metals.

[55] Wagner A., Bergner F., Chaouadi R., Hein H., Hernandez-Mayoral M., Serrano M., Ulbricht A., Altstadt E. (2016). Effect of neutron flux on the characteristics of irradiation-induced nanofeatures and hardening in pressure vessel steels. Acta Mat..

[56] Robertson C. WP4 overview: Irradiation tolerance. Proceedings of the INNUMAT European Project Final Symposium.

[57] Robertson C., Terentyev D., Gaganidze E., Chang C. (2026). Dose-dependent irradiation embrittlement in tungsten: A predictive model based on grain plasticity mechanisms. Int. J. Refract. Met. Hard Mat..

[58] Margolin B., Sorokin A., Pirogova N., Toivonen A., Sefta F., Pokor C. (2020). Analysis of mechanisms inducing corrosion cracking of irradiated austenitic steels and development of a model for prediction of crack initiation. Eng. Fail. Anal..

[59] Pokor C., Brechet Y., Dubuisson P., Massoud J.-P., Averty X. (2004). Irradiation damage in 304 and 316 stainless steels: Experimental investigation and modeling. Part II: Irradiation induced hardening. J. Nucl. Mater..

[60] Pokor C., Massoud J.P., Wintergerst M., Toivonen A., Ehrnsten U., Karlsen W. Determination of the time to failure curve as a function of stress for a highly irradiated AISI 304 stainless steel after constant load tests in simulated PWR water environment. Proceedings of the Fontevraud 7.

[61] Conermann J., Shogan R., Fujimoto K., Yonezawa T., Yamaguchi Y. Irradiation effects in a highly irradiated cold worked stainless steel removed from a commercial PWR. Proceedings of the 12th International Conference on Environmental Degradation of Materials in Nuclear Power System—Water Reactors, TMS.

[62] Robertson C. Non-hardening RPV steel embrittlement modelling. Proceedings of the ENTENTE European Project, Final Symposium.

[63] Messina L., Nastar M., Olsen P. (2014). Exact ab-initio transport coefficients in bcc Fe-X (X = Cr, Cu, Mn, Ni, P, Si) dilute alloys. Phys. Rev. B.

[64] Messina L. (2015). Multiscale Modelling of Atomic Transport. Ph.D. Thesis.

